# Editorial: Connecting Form and Function: Recent Advances in Understanding Dendrite Morphogenesis and Plasticity

**DOI:** 10.3389/fncel.2022.867364

**Published:** 2022-03-14

**Authors:** Quan Yuan, Chun Han, Peter Soba

**Affiliations:** ^1^Dendrite Morphogenesis and Plasticity Unit, National Institute of Neurological Disorders and Stroke, National Institutes of Health, Bethesda, MD, United States; ^2^Weill Institute for Cell and Molecular Biology and Department of Molecular Biology and Genetics, Cornell University, Ithaca, NY, United States; ^3^Department of Molecular Brain Physiology and Behavior, LIMES Institute, University of Bonn, Bonn, Germany; ^4^Institute of Physiology and Pathophysiology, Friedrich-Alexander-Universität Erlangen-Nürnberg, Erlangen, Germany

**Keywords:** dendrite, remodeling, neural development, structural plasticity, synapse development, neurological disorders, circuit connectivity

Neurons establish their dendritic arbors through a series of steps, from initial coverage of target areas and elaboration of fine branches for full innervation, to experience-dependent remodeling during circuit maturation or rewiring. The integrity and functions of dendrites further need to be maintained throughout the lifespan of the organism while keeping a delicate balance between stability and plasticity. As each of these steps requires orchestration of numerous intracellular events and interactions with the extracellular environment, there is a high demand on the molecular and cellular machinery specialized for supporting neuronal dendrites. This Research Topic highlights the range of intrinsic and extrinsic mechanisms of dendrite development, maintenance, and structural plasticity, as well as molecular pathways required for each process, many of them linked to neurological disorders and neurodegeneration.

During dendrite development, organization of the cytoskeleton plays a pivotal role in the structural integrity, providing transport tracks and growth force. Of particular interest is the organization of microtubules in neurons, which is reviewed by Wilkes and Moore with a focus on the formation and organization of microtubule organizing centers (MTOCs) at different stages of dendrite morphogenesis and spatial domains. Many additional intrinsic factors are required for proper dendritic patterning including kinase signaling pathways (Nourbakhsh and Yadav) as well as proteostasis, i.e., the maintenance of functional protein levels through synthesis and degradation (Lottes and Cox). Despite this ever-growing insight into molecular mechanisms of dendrite development, new players keep emerging through ongoing work. A clonal screen by the Wang et al. identified 40 new genes involved in dendrite morphogenesis in *Drosophila* somatosensory neurons, revealing the importance of tubulin folding, Nogo signaling, RNA splicing, phosphoinositides, and glycosylation.

Besides the cell intrinsic machinery, extrinsic mechanisms define many aspects of dendrite patterning across organisms. Two themes by which extrinsic factors exert their functions are local regulation of cell-cell adhesion and global regulation of transcription. Lin et al. reviewed such extrinsic factors discovered in a broad range of model systems that feature diverse spatial organization of dendritic arbors. Recent research also highlights the emerging importance of neuronal interaction with other tissues. For example, the epidermal cells that interact with somatosensory neurites are much more than passive bystanders: they actively promote neurite growth, position neurites in a 2-dimensional space or ensheathment and engulf pruned neurites. In this topic, Yin et al. specifically reviewed studies related to innervations of the epidermis by somatosensory neurons, while comparing findings in worm, fly, and zebra fish systems. Bridging intrinsic and extrinsic mechanisms, Shrestha et al. discovered that the immunoglobulin molecule Basigin is required in both neurons and surrounding epidermal cells, highlighting the importance of adhesion molecules on both growing dendrites and their supporting tissue.

While specific genetic programs orchestrate dendrite development and patterning, their final shape is strongly influenced by sensory experience and neural activity. The dynamic nature of the postsynaptic apparatus, spines, and dendritic arbors is a critical component of neuronal plasticity and has captivated neuroscientists over the past few decades. In this Research Topic, a review article by Furusawa and Emoto discussed several aspects of dendrite remodeling during development and injury across model systems, providing an overview of the recent progress made in this field.

Furthermore, new approaches and systems to study dendrite plasticity and the underlying molecular mechanisms are featured here, revisiting some of the most fundamental questions regarding dendritic structural plasticity. A classic example of activity-dependent dendrite remodeling is the dendritic pruning of mitral cells, which extend dendritic branches to multiple glomeruli that are thought to be trimmed down by odor-evoked activity after birth to contact only one specific glomerulus. Togashi et al. developed an Adeno-associated-virus (AAV)-based strategy to label developing mitral cells in the mouse olfactory bulbs independent of their birthdates. Surprisingly, they found that ~50% of mitral cells already completed their dendritic refinement to a single glomerulus by birth, suggesting that developmental mechanisms or spontaneous activity within the olfactory bulb play a major role in dendritic pruning of mitral cells.

Neuronal activity is not only a major driving force of developmental pruning, but is also required for synaptic plasticity, which is best studied in excitatory cortical neurons. Kuhlmann et al. used a cortical-striatal co-culture system to study activity-dependent plasticity in inhibitory Spiny Projection Neurons (SPNs). Both silencing glutamatergic inputs and chemically inducing NMDA receptor-dependent long-term-potentiation led to changes in spine density in a time-dependent fashion. These findings illustrate that inhibitory SPN plasticity can be induced by glutamate activity in the absence of dopamine and other neuromodulators, offering an experimental platform to be exploited in future studies. While glutamate-dependent synaptic plasticity and the responsible receptors have been investigated extensively, far less is known about the roles of nicotinergic acetylcholine receptors (nAchR), the major receptors for acetylcholine. Rosenthal and Yuan discussed the current understanding of *Drosophila* nAchRs, the best studied representatives of the predominant excitatory neurotransmitter receptor family in insects. The review highlighted decades of work on nAchRs' molecular features, as well as their critical functions in mediating short and long-term structural and functional plasticity. The technical advances made recently will likely improve our understanding on the function of cholinergic neurotransmission in dendrite development and plasticity across species.

Activity-dependent dendritic plasticity is a key feature underlying anatomical and functional changes in neuronal networks that likely also involve homeostatic mechanisms. In *Drosophila* motoneurons, Dhawan et al. identified several reactive oxygen species (ROS) signaling components as essential regulators for homeostatic structural plasticity of dendrites, and proposed a model on how this pathway and its downstream effectors regulate dendrite development in response to changes of synaptic activity. These findings create opportunities for additional mechanistic studies and further validation in other systems.

Because the molecular machinery governing dendrite development and plasticity is extremely complex, its components are often affected during aging and in neurological and neurodegenerative diseases. Several reviews in this series highlight key cellular and molecular processes of disorders affecting the integrity of dendrites. Here, Nourbakhsh and Yadav discussed the impact of kinase signaling on dendritic development and its connections to neurodevelopmental and neurodegenerative diseases. With promising novel techniques delineating their precise signaling pathways, kinases are emerging key players in neurological disorders. Additionally, injury-related kinase signaling pathways play a significant role in localized degeneration and regeneration (Furusawa and Emoto).

Besides specific signaling pathways, global mechanisms have a profound impact on preserving dendritic homeostasis. For example, dysregulated protein synthesis is strongly linked to Autism as well as neurodegenerative conditions. Lottes and Cox discussed the importance of maintaining proteostasis on the regulation of structural and functional integrity of dendrites. An equally profound impact on dendrite maintenance can be attributed to pathways regulating plasma membrane turnover. The review by Lin et al. summarized recent findings demonstrating the critical role of the secretory pathway and the exo- and endocytotic machinery in dendritic integrity. Due to their heightened vulnerability toward perturbations affecting plasma membrane turnover, dendrites are also the prime targets during neurodegeneration (Lin et al.).

In summary, this Research Topic reflects many recent progresses made in different model systems and with updated technologies, providing cellular and molecular insights into the making and breaking of neuronal dendrites. At the same time, these studies highlight the complexity and diversity of dendrites and remind us that much remains to be discovered.

## Author Contributions

All authors contributed equally to manuscript drafting, revision, reading, and approved the submitted version.

## Funding

This work was supported by the Intramural Research Program of National Institutes of Health (Project number 1ZIANS003137 to QY), National Institutes of Health (Grant number R01NS099125 to CH), German Research Foundation (DFG SPP1926, SO 1337/2-2, SO 1337/4-1, and SO 1337/7-1), the DFG Heisenberg Program (SO 1337/6-1), and ERA-NET NEURON (BMBF, 01EW1910 and 01EW2108 to PS).

## Conflict of Interest

The authors declare that the research was conducted in the absence of any commercial or financial relationships that could be construed as a potential conflict of interest.

## Publisher's Note

All claims expressed in this article are solely those of the authors and do not necessarily represent those of their affiliated organizations, or those of the publisher, the editors and the reviewers. Any product that may be evaluated in this article, or claim that may be made by its manufacturer, is not guaranteed or endorsed by the publisher.

